# Dietary phytochemical index in relation to risk of glioma: a case-control study in Iranian adults

**DOI:** 10.1186/s12937-021-00689-2

**Published:** 2021-04-01

**Authors:** Somaye Rigi, Mehdi Shayanfar, Seyed Mohammad Mousavi, Minoo Mohammad-Shirazi, Giuve Sharifi, Ahmad Esmaillzadeh

**Affiliations:** 1 Department of Community Nutrition, School of Nutritional Sciences and Dietetics, Tehran University of Medical Sciences, P.O. Box 14155-6117, Tehran, Iran; 2 Department of Clinical Nutrition and Dietetics, National Nutrition and Food Technology Research Institute, Shahid Beheshti University of Medical Sciences, Tehran, Iran; 3 Students’ Scientific Research Center (SSRC), Tehran University of Medical Sciences, Tehran, Iran; 4 Department of Neurosurgery, Loghman Hakim Hospital, Shahid Beheshti University of Medical Sciences, Tehran, Iran; 5 Obesity and Eating Habits Research Center, Endocrinology and Metabolism Molecular-Cellular Sciences Institute, Tehran University of Medical Sciences, Tehran, Iran; 6 Department of Community Nutrition, Food Security Research Center, Isfahan University of Medical Sciences, Isfahan, Iran

**Keywords:** Dietary phytochemical index, DPI, Diet quality, Glioma, Brain tumor

## Abstract

**Background & aim:**

No study is available that explores the association of dietary phytochemical index (DPI) with glioma. The objective of the current study was to assess this association in Iranian adults.

**Methods:**

This hospital-based case-control study included 128 newly-diagnosed cases of glioma and 256 age- and sex-matched controls. Data collection on dietary intakes was done using a 123-item validated food frequency questionnaire. Calculation of DPI was done as (dietary energy derived from phytochemical-rich foods (kcal)/total daily energy intake (kcal)) × 100. Logistic regression models were used to examine the association between DPI and glioma.

**Results:**

Individuals in the top tertile of DPI were more likely to be older and female. Before taking potential confounders into account, subjects in the top tertile of DPI tended to have a 40% reduced chance of glioma than those in the bottom tertile (OR: 0.60; 95% CI: 0.35–1.02, *P* = 0.06). After controlling for age, sex, energy intake, several demographic variables and dietary intakes, the association between DPI and glioma became strengthened (OR: 0.43; 95% CI: 0.19–0.97, *P* = 0.04).

**Conclusion:**

High intakes of phytochemical-rich foods were associated with a lower risk of glioma in adults. High consumption of phytochemical-rich foods might be recommended to prevent glioma. However, further studies with a prospective design are needed to confirm our findings**.**

## Background

Glioma, the most prevalent brain tumor, refers to all tumors that are supposed to originate from neuroglial cells [1]. About 77% of all brain malignant tumors are attributed to glioma [2]. The estimated incidence rate of brain tumors is 3.7 per 100,000 for men and 2.6 per 100,000 for women globally [3]. A mortality rate of 2.92 per 100,000 in men and 2.46 per 100,000 in women has been reported by a national study in Iran [4]. Due to the invasive nature and difficulty in removing affected areas, glioblastoma (which accounts for 45% of all gliomas) is known as cancer with the most inferior survival rate among various cancers [5]. Given the high mortality rate of brain malignancies, prioritizing the identification of contributing factors to the incidence and development of glioma is essential [6].

Earlier studies have reported some risk factors for glioma. For instance, farming was considered a high-risk job for glioma [7] because farmers do not wash their bodies immediately or do not take off the clothes after handling pesticides. In addition, residential places close to electromagnetic fields, broadcast and cell phone antennas over the last ten years were also reported as high-risk regions for the incidence of glioma [8]. Diet is a modifiable contributing factor to the abnormal proliferation and transformation of neuroglial cells [9, 10]. Epidemiologic studies on some food groups, including fresh fruits, vegetables, nuts and legumes in relation to glioma, have yielded protective associations [11–13]. These plant-based foods are rich in antioxidants, fiber and phytochemicals [14]. Flavonoids, phenolic acids, indoles, glucosinolates, phytoestrogens and isothiocyanates are the main phytochemicals (non-nutritive bioactive compounds) [15, 16], that were linked with cancer risk reduction [17]. In addition, the modulatory effect of phytochemicals on glioma’s degree of aggressiveness has been shown by several investigations [18]. Due to health promotional effects attributed to phytochemicals, measurement of dietary phytochemical quantity was proposed. Since the determination of diet’s phytochemical content was infeasible, the concept of the dietary phytochemical index (DPI) was suggested by McCarty. DPI is calculated via a simple method defined as the percentage of calorie intake derived from foods rich in phytochemicals [19]. This index has been proposed as an indicator of total phytochemical content of the diet and an index of better diet quality [20, 21]. Earlier studies have linked DPI to several chronic diseases. For instance, diets with a high DPI were inversely associated with obesity, oxidative stress, hypercholesterolemia, insulin resistance, hypertension and breast cancer [20, 22–25]. With respect to diet-glioma relations, although several dietary determinants have been reported, limited information are available about the association of DPI with this condition. Considering the inverse association between phytochemical-rich foods with glioma in earlier studies [26–28], we hypothesized that DPI might be associated with brain tumors. Therefore, we aimed to assess DPI in relation to the risk of glioma in the framework of a case-control study in Iranian adults.

## Methods

### Study design and subjects

Detailed information about study design, inclusion and exclusion criteria of this hospital-based case-control study have been reported previously [29]. Briefly, among 235 newly diagnosed pathologically confirmed glioma patients, the following cases were not included: 25 cases due to not meeting the inclusion criteria, 30 cases due to having severe form of glioma and disability, 22 cases due to their avoidance to cooperate and 30 cases due to defective medical information. Finally, 128 patients, including 75 men and 53 women aged between 20 and 75, were included. Among outpatients or admitted patients to the orthopedic and reconstructive surgery wards, 256 subjects, including 150 men and 106 women aged between 20 and 75, met our inclusion criteria. Included subjects were younger, leaner and also had higher education compared with excluded volunteers. Finally, we enrolled 128 cases and 256 matched (in the term of age and sex) controls between November 2009 and September 2011 in Tehran, Iran. The hospitals affiliated with Shahid Beheshti University of Medical Sciences were chosen as sampling sites using the convenience sampling method. Patients referring to the Neurosurgery department of the hospital, who had met our inclusion criteria, were regarded as cases. Individuals with pathologically confirmed glioma in the first month following detection (ICD-O-2, morphology codes 9380e9481), who were aged within the range of 20–75 years, were recruited. Controls were chosen from apparently healthy individuals (aged within the range of 20–75) who had been referred to other wards (orthopedic or surgery wards) of the same hospital. All cases and controls completed an informed consent form before data collection initiation. The main project on glioma was first ethically approved by the Iran National Nutrition and Food Technology Research Institute in 2009, before the study (Ethical code: 39414). Then, based on the main dataset, other projects were defined, each with a different exposure. Mostly, each of these projects that were later written based on that dataset has its own approvals. For the current study, the Medical Ethics Committee of Tehran University of Medical Sciences ethically approved the study (2020/06/22, Ethical code, IR.TUMS.MEDICINE.REC.1399.162).

### Inclusion and exclusion criteria

Qualified cases for taking part in our project were those who met the following inclusion criteria (1) individuals with pathologically confirmed glioma with a maximum one-month interval after diagnosis of glioma, (2) aged between 20 and 75 years old. Controls were healthy subjects that had the same age and sex as cases.

**Fig. 1:**
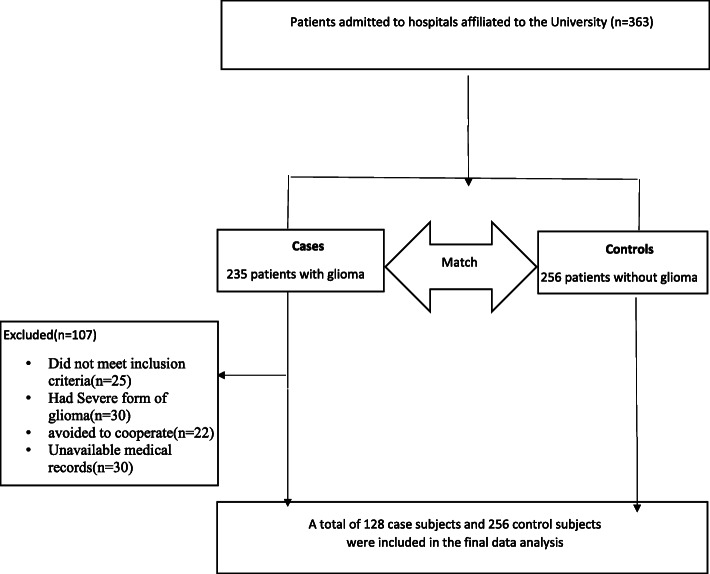
Flow diagram showing the study recruitment process

The following items were regarded as exclusion criteria: (1) being pregnant or lactating, (2) having a history of some disorders including cancer (only in control groups), neurological, gastrointestinal, hepatic, endocrine, immune, kidney and cardiovascular diseases in medical records, (3) being on special diets, which might result in changes in routine dietary intakes, (4) having any history of chemotherapy or radiation therapy, and (5) use of nitrosamine-enhancing drugs (Fig. 1).

### Dietary intake assessment

In this study, trained interviewers administered a Block-format-validated 123-item semi-quantitative food frequency questionnaire (FFQ) to evaluate dietary intakes of subjects over the past year [30]. Each participant reported his/her average intakes of different food items (per day, week or month) in a face-to-face interview. Considering the U.S. Department of Agriculture’s food composition database (modified for Iranian foods) [31], daily nutrients and energy intakes were estimated using Nutritionist IV software (First Databank, Hearst Corp., SanBruno, CA, USA). A validation study [17] revealed reasonable estimates of long-term dietary intakes for this questionnaire because good correlations were seen between dietary intakes obtained from this questionnaire and those from the average of 24-h dietary recalls (two recalls in each month of a year) as the gold standard. For example, energy-adjusted correlation coefficients for vitamin C, vitamin E and b-carotene were estimated as 0.65, 0.65, and 0.68, respectively [16, 18].

### Calculation of dietary phytochemical index

We estimated DPI using McCarty equation [10]:

$$ (DPI)=\frac{dietary\kern0.34em energy\kern0.34em derived\kern0.34em from\kern0.34em phytochemical- rich\kern0.34em foods\kern0.28em (kcal)}{total\kern0.34em daily\kern0.34em energy\kern0.17em \mathrm{intake}\kern0.17em (kcal)}\mathrm{X}100 $$

The phytochemical-rich foods we considered in the current study were as follows: Whole grains (Sangak and Barbari bread, which are traditional Iranian breads); fruits (red, yellow and orange fruits); vegetables (dark green vegetables, red, orange vegetables, starchy vegetables and other vegetables); soy products (soybean); nuts (peanut, almond, walnut, pistachio and hazelnut); legumes (lentil, beans, chickpea); olives; olive oil; natural fruit and vegetable juices (carrot juice, orange juice, Limon juice). Potato, as a food item in the vegetable group, was not considered in DPI calculation due to its low content of phytochemicals.

### Assessment of glioma

Detection of glioma was performed based on the pathological test ICD-O-2 and morphology codes 9380–948 [29]. Glioma patients who had passed a  maximum of one month of the disease confirmation were included in our study.

### Assessment of other variables

A pretested questionnaire including several variables of sociodemographic status such as age (years), gender (male/female), the status of marriage (married/unmarried), residence place (urban/rural), occupation (farmer/non-farmer), education (university graduated/non-university graduated) and family history of glioma and any other cancer (yes/no), a history of trauma, hypertension and allergy (yes/no), dealing with chemicals during the past ten years (yes/no), methods of cooking (barbecue/microwave/canned foods/fried foods), drug use (yes/no), use of hair dye (yes/no), cell phone use duration (years), exposure to the radiographic x-ray (yes/no); was applied to collect general information of participants. Measurement of participants’ physical activity was done using a short form of the International Physical Activity Questionnaire (IPAQ). Data from IPAQ were stated as Metabolic Equivalent per week (METs/week). Anthropometric measurements were quantified via standard methods. Considering weight and height, body mass index (BMI) was calculated for each participant.

### Statistical analysis

All subjects were classified based on the DPI score into tertile ranges. The distribution of study participants in terms of general characteristics across tertiles of DPI was assessed using the Chi-square test. Differences in continuous variables across DPI tertiles were determined using one-way analysis of variance (ANOVA), followed by pairwise post hoc tests with Bonferroni correction. Binary logistic regression was used to evaluate the association of DPI with glioma. Age (continuous), sex (male/female), energy intake (kcal/day), physical activity (continues), family history of cancers (yes/no), family history of glioma (yes/no), marital status (yes/no), education (university graduated/ non-university graduated), high-risk occupation (farmer/non-farmer), high-risk residential area (yes/no), duration of cell phone use (continues), supplement use (yes/no), history of exposure to the radiographic X-ray (yes/no), history of head trauma (yes/no), history of allergy (yes/no), history of hypertension (yes/no), smoking status (smoker/non-smoker), exposure to chemicals (yes/no), drug use (yes/no), personal hair dye (yes/no), frequent fried food intake (yes/no), frequent use of barbecue (yes/no), canned foods and microwave (yes/no), dietary intakes of red and processed meat, fish, tea, coffee, sugar-sweetened beverages, egg, total fat, dietary fiber, cholesterol, calcium, SFA, folate, and selenium were adjusted in the multivariable-adjusted model. The selection of these confounders was made based on previous publications [11, 32–34]. The model goodness-of-fit was examined using Hosmer–Lemeshow test. By considering tertiles of DPI as ordinal variables, the overall trend of ORs across increasing tertiles of DPI was examined. All the statistical analyses were performed using SPSS (SPSS Inc., version 19). The significance of *P*-values was considered at < 0.05.

## Results

Table 1 shows the percentage of subjects consuming adequate nutrient intakes from food and supplements. All participants in both case and control groups met RDA for protein and carbohydrate (100%). However, inadequate intake was likely for 10 of 12 evaluated macronutrients, vitamins and minerals. Although most participants were likely to have adequate intakes of dietary fiber, SFA, vitamin B6, vitamin C, and potassium, less than 30% of participants did not meet the DRIs for these nutrients. In addition, the majority of participants did not meet adequate intakes of vitamin E. Less than half of female cases and controls had adequate intakes of calcium, while more than half of male cases and controls had adequate intakes of this nutrient. More than 90 % of female cases and controls did not meet the DRI for folate. The corresponding percentage for male controls was 67.8% and for male cases was 69.3%.

**Table 1:** Adequate intake of selected nutrients in cases and controls separately for men and women

	Criteria for adequate intake		Groups					
	RDA		Controls (*n = 256*)			Cases (*n = 128*)		
	Males	Females	Males	Females	P^*^	Males	Females	P^*^
Protein (%)	56	46	99.3	100	0.38	100	100	0.99
Carbohydrate (%)	130	130	100	100	0.99	100	100	0.99
Dietary fiber (%))	38	25	87.5	87.9	0.88	86.7	84.9	0.77
SFA (%)	< 10% of calories ^a^	< 10% of calories ^a^	92.6	86	0.08	93.3	90.6	0.56
Calcium (%)	1000	1000	67.8	53.3	0.02	60	34	0.004
Vitamin E (%)	15	15	0.7	0.0	0.39	0.0	0.0	0.99
Vitamin B6 (%)	1.3	1.3	92.6	87.5	0.19	94.7	79.2	0.008
Folate (%)	400	400	32.2	9.3	< 0.001	30.7	7.5	0.002
Vitamin C (%)	90	75	86.6	89.7	0.58	80	88.7	0.04
Potassium (%)	3400	3400	82.6	77.6	0.32	86.7	64.2	0.003
Selenium (%)	55	55	58.4	29.9	< 0.001	69.3	35.8	< 0.001

Data are presented as percent

^*^Obtained from Chi-square test

^a^ Acceptable macronutrient distribution range (AMDR)

Cases and controls were not significantly different in terms of mean age, BMI and physical activity. The comparison of general characteristics of study participants across DPI tertiles revealed that individuals in the highest tertile were more likely to be physically active, use hair dye and drugs than those in the lowest tertile. A higher percentage of subjects in the highest category of DPI were married than those in the lowest category. Higher DPI was associated with older age and less frequent fried food intake. A lower percentage of individuals in the highest category of DPI were males. Subjects in the highest category of DPI had shorter cell phone use duration compared with those in the lowest category. No other significant differences were seen in terms of other general characteristics across tertiles of DPI (Table 2).

**Table 2:** General characteristics of study participants across tertiles of DPI

	Tertiles of DPI			
	T1 ≤21 *n = 128*	T2 21 < to< 30 *n = 128*	T3 ≥30 *n = 128*	P^*^
Age (years)	39 ± 14	42 ± 13	46 ± 13†	0.001
BMI (kg/m^2^)	26.3 ± 4	26 ± 3	26.1 ± 4	0.20
Males (%)	68	56.3	50.8	0.01
Married (%)	72.7	84.4	82	0.02
University graduated (%)	16.4	14.1	14.9	0.31
High-risk jobs ^a^ (%)	3.9	3.1	8.6	0.10
High-risk residential area ^b^ (%)	24.2	24.2	25	0.98
Duration of cell phone use (years)	3.8 ± 2.8	3.5 ± 2.4	2.5 ± 2.7†	0.04
History of exposure to the radiographic X-ray (%)	6.3	10.9	13.3	0.16
History of dental photography (%)	49.2	59.4	55.5	0.25
History of head trauma (%)	34.4	27.3	39.8	0.10
History of allergy (%)	21.9	29.7	32	0.16
History of hypertension (%)	2.3	3.9	6.3	0.29
Current smoker (%)	24.2	18	23.4	0.42
Frequent fried food intake ^c^ (%)	84.4	89.8	72.7	0.001
Frequent use of barbecue ^d^ (%)	16.4	15.6	7.8	0.08
Frequent microwave use (%)	18	18.8	9.4	0.07
Frequent canned foods intake (%)	8.6	3.1	6.3	0.18
Drug use (%)	3.9	3.1	10.9	0.01
Personal hair dye use (%)	22.7	39.1	42.2	0.002
Exposure to chemicals (%)	15.6	10.2	14.8	0.38
Family history of glioma (%)	9.4	13.3	7.8	0.32
Family history of cancer (%)	37.5	27.3	35.9	0.18
Supplement use (%)	13.3	12.5	13.3	0.97
Physical activity (METs)	32.8 ± 5.7	34 ± 5.2	35.5 ± 6.1†	0.001

Data are presented as mean ± standard deviation (SD) or percent

^a^ Farmers were considered as having a high-risk occupation

^b^ Persons who lived in places nearby electromagnetic fields and cell phone and broadcast antennas in the last 10 years were considered as living in high-risk areas

^c^ Persons who consumed fried food at least twice per week were considered as frequent fried food users

^d^ Persons who used barbecue, microwave and canned foods at least twice per week were considered as frequent users

^*^ Obtained from ANOVA with Bonferroni correction or Chi-square test, where appropriate

† *P*-value for the comparison with T1 < 0.05

Table 3 presents the dietary intakes of study participants across tertiles of DPI. Individuals in the top tertile of DPI had higher intakes of vegetables, fruits, legumes and whole grains, and they had lower intakes of dietary fiber, red and processed meats, cholesterol, folate, selenium, refined grains and sugar-sweetened beverage compared with those in the bottom tertile.

**Table 3:** Dietary and nutrient intakes of study participants across tertiles of DPI

	Tertiles of DPI			
	T1 ≤21 *n = 128*	T2 21 < to< 30 *n = 128*	T3 ≥30 *n = 128*	*P* ^a^
Energy (kcal/day)	2700 ± 843	2494 ± 554†	2508 ± 560	0.05
**Nutrient intakes**				
Protein (g/day)	101 ± 37	94 ± 20	96 ± 21	0.14
Fat (g/day)	67 ± 25	62 ± 17	64 ± 18	0.28
Carbohydrate (g/d)	436 ± 153	403 ± 98	409 ± 98	0.11
Dietary fiber (g/day)	27.1 ± 19.5	21.8 ± 8.7†	20.2 ± 6.2†	0.001
Cholesterol (mg/day)	275 ± 182	234 ± 82†	211 ± 86†	0.001
SFA (g/day)	21.5 ± 10	19.6 ± 7.8	19.2 ± 7	0.09
Calcium (mg/day)	1161 ± 431	1069 ± 259	1063 ± 272	0.08
Vitamin E (mg/day)	5.6 ± 2.3	5.1 ± 2.4	5.7 ± 2.6	0.13
Vitamin B6 (mg/day)	2 ± 1	1.8 ± 0.4	1.8 ± 0.4	0.11
Folate (mcg/day)	416 ± 416	348 ± 77	345 ± 77	0.04
Vitamin C (mg/d)	142 ± 160	135 ± 37	134 ± 40	0.78
Potassium (mg/d)	4399 ± 1854	4131 ± 725	4264 ± 821	0.20
Selenium (mg/d)	0.08 ± 0.5	0.06 ± 0.03†	0.05 ± 0.02†	< 0.001
**Food groups**				
Refined grains (g/day)	268 ± 120	210 ± 94†	125 ± 84†‡	< 0.001
Whole grains (g/day)	97 ± 109	131 ± 78†	248 ± 105†‡	< 0.001
Red and processed meats (g/day)	41 ± 31	37 ± 17	34 ± 17	0.01
Poultry (g/day)	34 ± 30	31 ± 12	29 ± 11	0.12
Vegetables(g/day)	247 ± 82	274 ± 78†	283 ± 91†	0.002
Fruits (g/day)	327 ± 120	357 ± 108	361 ± 121†	0.04
Fish (g/day)	9.6 ± 13	8.2 ± 8	9.5 ± 9	0.40
Nuts (g/day)	4.6 ± 4.5	4.3 ± 3	4.6 ± 3.7	0.79
Legumes (g/day)	32 ± 17	34 ± 18	38 ± 20†	0.01
Sugar-sweetened beverage (g/day)	95 ± 82	81 ± 59	68 ± 70†	0.02

Data are presented as mean ± SD

^a^ Obtained from ANOVA with Bonferroni correction

† P-value for the comparison with T1 < 0.05

† P-value for the comparison with T2 < 0.05

Crude and multivariable-adjusted ORs for glioma across tertiles of the DPI are presented in Table 4. Before taking potential confounders into account, subjects in the top tertile of DPI tended to have a 40% reduced chance of having glioma than those in the bottom tertile (OR: 0.60; 95% CI: 0.35–1.02, *P* = 0.06). After controlling for age, sex, energy intake, several demographic variables and dietary intakes, the association between DPI and glioma became strengthened (OR: 0.43; 95% CI: 0.19–0.97, *P* = 0.04). The Hosmer-Lemeshow goodness-of-fit test suggested an excellent calibration for the multivariable regression model (Chi-square = 11.06; degrees of freedom = 8; *P* = 0.20).

**Table 4:** Odds ratios (ORs) and 95% confidence intervals (95% CIs) of glioma according to tertiles of DPI*

	Tertiles of DPI			*P*_trend_^*^
	T1	T2	T3	
DPI scores	≤21	21 < to< 30	≥30	
No. of participants	*n = 128*	*n = 128*	*n = 128*	
Crude	1.00	0.93 (0.56–1.55)	0.60 (0.35–1.02)	0.06
Multivariable-adjusted^a^	1.00	0.77 (0.39–1.54)	0.43 (0.19–0.97)	0.04

*Binary logistic regression was used to obtain OR and 95% CI. The overall trend of OR across increasing tertiles was examined by considering each category’s median score as a continuous variable

^a^ Adjusted for age, sex, and energy intake, physical activity, family history of cancer, family history of glioma, marital status, education, high-risk residential area, duration of cell phone use, supplement use, history of exposure to the radiographic X-ray, history of head trauma, history of allergy, history of hypertension, smoking status, exposure to chemicals, drug use, personal hair dye use, frequent fried food intake, frequent use of barbecue, canned foods and microwave, red and processed meat, fish, tea and coffee and, sugar-sweetened beverage, egg, total fat, dietary fiber, cholesterol, folate, selenium

Hosmer-Lemeshow goodness-of-fit test: Chi-square = 11.06; degrees of freedom = 8; P = 0.20

## Discussion

Our findings from this hospital-based case-control study revealed an inverse association between DPI and the odds of glioma after adjusting for a wide range of potential confounders. Although several studies have suggested fruits, vegetables, nuts and legumes as prophylactic agents with respect to the diet-glioma link [10, 35–37], to the best of our knowledge, this work is the first investigation that assessed the association between DPI and glioma.

Because nearly 90 % of glioma patients die within three years after detection, it stands among the most lethal malignancies in the world [38]. Therefore, finding preventive measures is of high priority. Dietary factors play a pivotal role in the incidence and development of glioma [39]. Dietary indices, that reflect diet quality, have frequently been used to investigate diet-disease relations. It seems that DPI can also be considered as an indicator of better diet quality [40].

In the current study, subjects in the top tertile of DPI had 57% lower odds of glioma than those in the bottom tertile. Food groups that are considered phytochemical-rich sources have previously been investigated regarding glioma risk [39, 41, 42]. For instance, dietary intake of whole grains, fruits, vegetables, legumes and nuts have been assessed in relation to glioma [39, 41, 42]. In line with our findings, a case-control study on the link between the DASH-style eating pattern (which is rich in fruits, vegetables, plant proteins from nuts and legumes) and risk of glioma reported that individuals with the greatest adherence to the DASH diet were 72% less likely to have glioma compared with those with the lowest adherence [41]. Also, the Mediterranean diet, which is characterized by high intakes of vegetables, legumes, fruits, whole grains and olive oil, has been assessed in relation to the risk of cancer in a systematic review and meta-analysis of observational studies; such that the highest adherence to the Mediterranean diet was associated with a lower risk of several cancers. However, no significant association was found for esophageal/ ovarian/ endometrial and bladder cancer [43]. Findings from a recent meta-analysis suggested an inverse association between vegetable intake and the risk of glioma [42]. In that analysis, a protective association was also reported between fruit consumption and glioma in Asian population, but not among white population [42]. There was an inverse association between whole-grain intake and total cancer [44]. Finally, an inverse association was found between legume consumption and risk of colorectal cancer in a meta-analysis on prospective cohort studies [45]. Overall, it seems that all foods with a high content of phytochemicals might help to prevent cancer incidence, including glioma.

Various mechanisms have been hypothesized linking phytochemical-rich foods with glioma. Anticancer property has been attributed to the main phytochemicals in these sources. It is well-known that oxidative stress is a process by which produced reactive oxygen species take part in glioma pathogenesis and antioxidant intake can prevent the development of glioma [39]. Phytochemicals as the main ingredients of the diet with antioxidant property [46] may exhibit favorable effects in this regard. In addition, phytochemicals take part in health and physiology function through anti-inflammatory potential and as an anti-proliferative agent for initiated and transformed cells modulate critical cellular signaling pathways [47, 48].

Several strengths of the present study worth noting. To the best of our knowledge, this study is the first to examine the association between DPI and glioma risk. Adjustment for a wide range of potential confounders indicated an independent link between DPI and odds of glioma. In addition, cases were selected from newly-diagnosed glioma patients due to the possibility of altering their dietary habits after glioma detection. Finally, previous investigations proved the effect of energy intake on all foods and nutrients; therefore, energy-adjusted DPI was used to remove energy from other foods and nutrients. This can help reducing misclassification of study participants in terms of DRI. Limitations of our study include the following: (a) lack of containing all the dietary items of phytochemical-rich foods like spices in the DPI calculation, (b) the inherent limitations in the calculation of DPI; for instance, non-caloric phytochemical-rich foods like green and black tea were not included in the calculation. In addition, lack of differentiation between consumed phytochemicals’ type in DPI score, to elaborate, a diet with more legumes and nuts may have the same DPI score as a diet with more fruits and vegetables; therefore, the various quality of dietary phytochemicals among people with the same DPI score affects the risk of glioma differently, (c) the U.S. Department of Agriculture FCT was applied to calculate energy and nutrient intakes due to incompetence Iranian FCT, (d) the nature of case-control design with its inherent possibility of selection and recall bias would not allow us to confer causality, (e) using FFQ for dietary assessment results in misclassification which is unavoidable in epidemiologic studies, (f) lack of the use of molecular parameters along with tumors’ morphology to identify the tumors, and (g) as eating habits of Iranians are different from other countries in the world, the generalizability of our findings must be done cautiously. Although the results cannot be extrapolated to populations of other countries, it must be kept in mind that phytochemicals exist in various dietary sources. Based on the current study’s findings, taking these dietary components from any food source might help prevent glioma even in other populations.

## Conclusion

In conclusion, this case-control study’s findings indicate that a diet with a high amount of phytochemical-rich foods was associated with a lower odds of glioma in Iranian adults. Although our findings support the current recommendations of consumption of phytochemical-rich foods in the daily diet, it seems that adherence to popular diets that include high amounts of fruit and vegetables, including the Mediterranean diet or the vegetarian diets with low refined grains, potato products, hard liquors, added sugars and oils might help preventing glioma. Of note, the DPI of most current diets, especially in developed countries, would be unlikely to be as high as 20, which means that there would be quite ample room for improvement. Considering that such modification would result in increased intakes of potassium, fiber, vitamins, trace minerals and plant proteins and increase dietary phytochemical intake, the contribution of such diets to human health would be enormous.

## Data Availability

The datasets used and/or analyzed during the current study are available from the corresponding author on reasonable request**.**

## Abbreviations

DPI: Dietary phytochemical index
FFQ: Food frequency questionnaire
IPAQ: International Physical Activity Questionnaire
BMI: Body mass index
ANOVA: One-way analysis of variance
